# Association Between High-Sensitivity C-Reactive Protein Levels and Metabolic Dysfunction-Associated Steatotic Liver Disease and Liver Fibrosis Risk: A Study Based on NHANES Data

**DOI:** 10.5152/tjg.2025.24251

**Published:** 2025-06-23

**Authors:** Chaolong Xu, Kai Wang, Zhiming Peng, Peng Chen, Chengchen Zhang, Xianyi Zeng, Chenghao Tan, Yunchen Huang

**Affiliations:** 1 Department of Hepatobiliary Surgery, People’s Hospital of Yicheng City, Yicheng, China

**Keywords:** High-sensitivity C-reactive protein, liver fibrosis, metabolic dysfunction-associated steatotic liver disease, NHANES, risk assessment, transient elastography

## Abstract

**Background/Aims:**

High-sensitivity C-reactive protein (hs-CRP) is a known inflammatory biomarker linked to various metabolic disorders. This study sought to examine the association between hs-CRP levels and the risk of metabolic dysfunction-associated steatotic liver disease (MASLD) and liver fibrosis (LF).

**Materials and Methods:**

Data from 2787 participants of the 2017-2020 National Health and Nutrition Examination Survey were analyzed. The evaluation of liver steatosis and fibrosis was performed using transient elastography. Furthermore, logistic regression models were applied to examine the correlation between 4 categorized levels of hs-CRP and the risks of MASLD and LF.

**Results:**

Compared to individuals with hs-CRP ≤3 mg/L, those with hs-CRP levels of 3.01-6 mg/L, 6.01-10 mg/L, and ≥10.01 mg/L exhibited markedly increased risks of MASLD, with odds ratios and 95% CI of 2.229 (1.892-2.625), 2.474 (1.982-3.090), and 3.175 (2.497-4.037), respectively. The receiver operating characteristic and calibration curves of the model validated the significant association of higher hs-CRP levels with increased MASLD and LF risk.

**Conclusion:**

Elevated hs-CRP levels are prominently associated with increased risks of MASLD and LF. High-sensitivity C-reactive protein could serve as a potential biomarker for identifying and managing MASLD and related fibrosis risks.

**Main Points**

- Elevated high-sensitivity C-reactive protein (hs-CRP) levels are significantly associated with an increased risk of metabolic dysfunction-associated steatotic liver disease (MASLD) and liver fibrosis (LF).
- The hs-CRP concentrations exceeding 10.01 mg/L indicate a heightened MASLD and fibrosis risk.
- The study confirms hs-CRP as a potential biomarker for early detection and risk assessment of MASLD and LF.
- Utilizing large-scale National Health and Nutrition Examination Survey data, the research provides robust evidence linking systemic inflammation to liver disease progression.
- The findings suggest the integration of hs-CRP measurement in routine clinical screening for MASLD and related fibrosis.

## Introduction

Non-alcoholic fatty liver disease (NAFLD) is widely recognized in clinical practice as closely associated with obesity, diabetes, hypertension, and dyslipidemia, collectively known as metabolic syndrome.^1^^,^^2^ Notably, NAFLD has been reclassified as metabolic dysfunction-associated fatty liver disease (MAFLD).^3^ Following this reclassification, NAFLD has again been redefined as MASLD.^4^ Metabolic dysfunction-associated fatty liver disease represents a globally prevalent metabolic condition characterized by excessive hepatic fat accumulation devoid of significant alcohol consumption history. The worldwide prevalence of MASLD has surged to 30%, with a continuous upward trajectory.^5^ In some cases, NAFLD may progress to non-alcoholic steatohepatitis, cirrhosis, and even hepatocellular carcinoma.^6^

High-sensitivity C-reactive protein (hs-CRP), an acute-phase protein, is pivotal in systemic inflammatory responses.^7^ As inflammation sets in, hs-CRP levels significantly rise, making it a widely employed biomarker in clinics for inflammation and cardiovascular risk.^8^ Recent studies suggest that beyond the established association with cardiovascular diseases, hs-CRP may also be implicated in the pathogenesis of MASLD.^9^^,^^10^ While some research indicates that serum hs-CRP concentrations robustly predict MASLD^11^ and can serve as an indicator of MASLD hepatic damage,^12^ other studies argue that hs-CRP cannot reliably predict the severity of MASLD.^13^^,^^14^

However, a significant research gap persists regarding the association between hs-CRP, MASLD, and liver fibrosis (LF). While some studies hint at a possible relationship, they often involve small sample sizes or are confined to specific populations, leaving a void in large-scale, diverse population studies. Clarifying this association holds considerable clinical implications for risk assessment and the formulation of preventive and therapeutic strategies.

This study analyzed data from the National Health and Nutrition Examination Survey (NHANES) spanning 2017-2020. Transient elastography was utilized to assess fatty liver levels and fibrosis in participants. Binary logistic regression models were employed to investigate the potential association between hs-CRP levels, MASLD, and LF. These methods were chosen for their reliability and accuracy in similar studies. The study aimed to address the current research gap concerning the relationships among hs-CRP, MASLD, and LF and offer an additional biological rationale for risk assessment and treatment. Based on NHANES data, the study sought to enhance early diagnosis and offer new insights for personalized treatment strategies targeting both MASLD and LF.

## Materials and Methods

### Study Participants

This study was a retrospective analysis. Liver ultrasound transient elastography data from the NHANES database (from 2017 to 2020) were analyzed in this study. National Health and Nutrition Examination Survey, a representative survey reflecting the health and nutritional status of the U.S. population, was approved by the National Center for Health Statistics Research Ethics Review Board and ensured informed consent from all participants. As NHANES data is publicly available and de-identified, no additional ethical approval was required for the present study. Detailed methodologies and data collection processes of NHANES have been comprehensively outlined on their official website (https://wwwn.cdc.gov/Nchs/Nhanes/Search/DataPage.aspx?Component=Examination&Cycle=2017-2020). In this study, relevant liver ultrasound elastography data were selected to investigate their associations and potential impacts on the health status of the general U.S. population.

### Inclusion and Exclusion Criteria

In the survey cycle, a total of 10 409 individuals completed the visit. Participants younger than 20 years and those who were pregnant or uncertain about their pregnancy status at the time of the examination were excluded. Individuals with missing hs-CRP data or incomplete liver ultrasound transient elastography examination data were excluded. Participants who tested positive for any markers of viral hepatitis (hepatitis B surface antigen, hepatitis D antibody, hepatitis C RNA, or hepatitis E IgM), those who consumed alcohol beyond the recommended limit of more than 1 drink per day for women and more than 2 drinks per day for men),^15^ or those with incomplete information on variables such as age, gender, education level, recreational physical activity, body mass index (BMI), smoking habits, diabetes, levels of total cholesterol, high-density lipoprotein cholesterol (HDL-C), aspartate aminotransferase (AST), and alanine aminotransferase (ALT) were also excluded from the study.

### Measurement of Hepatic Steatosis and Fibrosis through Ultrasonic Transient Elastography

Transient elastography, a liver ultrasound technique, provides a non-invasive and FDA (Food and Drug Administration)-approved method for accurately assessing the extent of steatosis and fibrosis in patients diagnosed with MASLD and is utilized for evaluating LF. The FibroScan® device employs ultrasound and vibration-controlled transient elastography to measure liver stiffness, concurrently assessing ultrasound attenuation related to hepatic steatosis. Controlled attenuation parameter (CAP) values are recorded as indicators of hepatic steatosis. Participants were included in the study if they had fasted for at least 3 hours before the examination, obtained 10 or more credible liver stiffness measurements (E), and achieved an interquartile range/median E ratio (IQRe) below 30%. Recent clinical practice guidelines issued by the European Association for the Study of the Liver on non-invasive methods for liver disease assessment^16^ demonstrate that a CAP value exceeding 275 dB/m is indicative of steatosis. Hence, MASLD in this study was characterized by a CAP score ≥ 275 dB/m. Additionally, significant fibrosis, corresponding to a grade of F2 or higher, was defined by a median liver stiffness value ≥ 8.2 kPa.^17^

### Selection and Definition of Categorical Variables

The following variables were included in the study as categorical variables: gender, race/ethnicity, educational level, recreational physical activity, BMI, smoking status, diabetes, and hs-CRP. High-sensitivity C-reactive protein (mg/L) was categorized into 4 groups: ≤ 3, 3.01-6, 6.01-10, and ≥ 10.01. Body mass index, calculated as weight/height^2^, was classified into 3 categories: underweight/normal, overweight, or obese (respectively <25, 25-29.9, ≥30 kg/m^2^). Educational level was divided into <high school, high school, and >high school. Race/ethnicity categories included Mexican American, other Hispanic, non-Hispanic white, non-Hispanic black, non-Hispanic Asian, and multi-racial races. Diagnosis of diabetes in participants was confirmed if they met one of the following conditions: (a) glycohemoglobin (%) of at least 6.5% or fasting plasma glucose levels exceeding 126 mg/dL; (b) positive responses to queries regarding a doctor’s diagnosis of diabetes, current insulin usage, or the use of oral diabetic medications to reduce blood sugar levels. The classification of smoking status was based on the response to whether participants had consumed 100 cigarettes or more in their lifetime. Those who replied “no” were identified as never smokers. Those answering “yes” were then further categorized, depending on whether they still smoked or had quit, into current smokers or former smokers, respectively.^18^

### Enhanced Logistic Regression Analysis and Model Validation Procedure

Data preprocessing was conducted to prepare for subsequent logistic regression analysis, including data cleaning, missing value analysis, outlier analysis, and conversion of categorical variables MASLD and fibrosis into binary variables. A logistic regression model incorporating multiple covariates was then constructed, with the model form specified as logit (P) = β0 + β1 × Race. Ethnicity + β2 × HDL cholesterol (mmol/L) + … + βk × AST(IU/L) (where β0 is the intercept, β1, β2, …, βk are the regression coefficients of each covariate), where P is the probability of having MASLD or fibrosis. To assess the model’s performance, the classification capability was measured through the receiver operating characteristic (ROC) curve and the area under the ROC curve (AUC) and used calibration curves to compare the model-predicted event occurrence probability with the actual observed probability. Based on the constructed model, the odds ratios (OR) and 95% CIs for MASLD or fibrosis across different hs-CRP categories were calculated, providing relative risk estimates compared to the reference category. Variable importance analysis was also conducted to identify the most influential variables in the model.

### Statistical Analysis

All statistical analyses were performed using R version 4.1.2. (R Foundation for Statistical Computing; Vienna, Austria) Given the complex design of NHANES, data analyses were appropriately weighted following the NHANES survey guidelines. Survey-weighted means (95% CI) were reported for continuous variables, and *P*-values were calculated using survey-weighted linear regression. Survey-weighted percentages (95% CI) were reported for categorical variables, and *P*-values were obtained through survey-weighted chi-square tests. For numerical variables with sample sizes not exceeding 5000, normality tests were conducted; based on the results, either mean ± standard deviation (SD) or median (interquartile range) was used to describe the data. For sample sizes exceeding 5000, mean ± SD was calculated directly. Statistical methods were chosen based on data normality and variance homogeneity: *t*-test, Welch’s *t*-test, one-way ANOVA, Welch’s one-way ANOVA, Wilcoxon test, or Kruskal–Wallis test for numerical variables; chi-square test, continuity correction chi-square test, or Fisher’s exact test for categorical variables. Normally distributed data were described using mean (SD) and compared using *t*-tests, while non-normally distributed data were described using median (interquartile range) and compared using Mann–Whitney *U* tests. Count data were expressed as numbers (percentages) and analyzed using chi-square tests. During model construction and validation, the caret and pROC packages in R were utilized, and all graphs were generated using ggplot2 and related plotting packages. A *P*-value of <.05 was considered statistically significant.

## Results

### Demographic and Clinical Characteristics in Metabolic Dysfunction-Associated Steatotic Liver Disease Patients and its Liver Fibrosis Subsets

Metabolic dysfunction-associated steatotic liver disease and its potential progression to LF have garnered considerable attention in the extensive field of liver diseases. This study examined the demographic and clinical differences between MASLD patients, their significant fibrosis subset, and non-MASLD participants to identify potential associations and risk factors.

**Figure 1.**
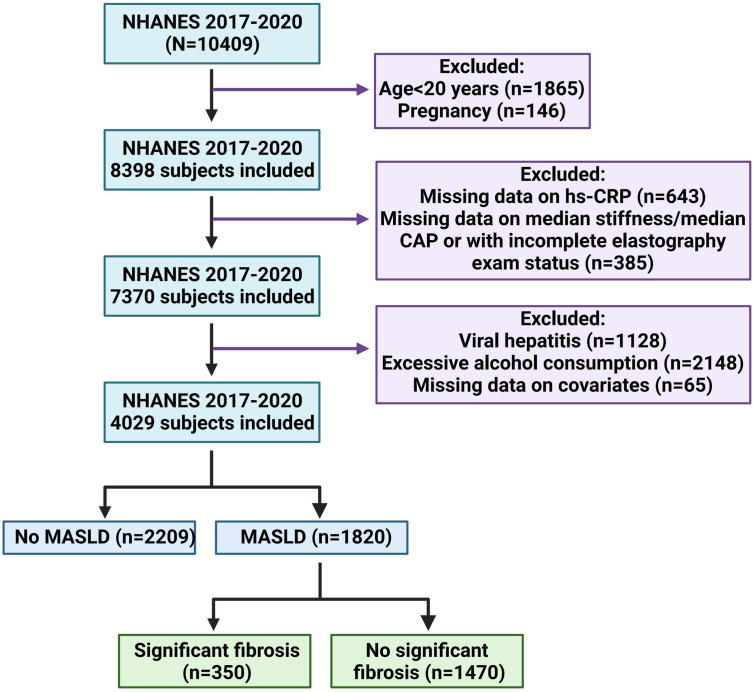
Flowchart of NHANES sample selection, 2017-2020.

A total of 4029 participants were included in this study, with 1820 confirmed cases of MASLD and 2209 non-MASLD cases. Among the MASLD patients, 350 exhibited significant fibrosis, while the remaining 1470 did not (Figure 1). Table 1 outlines the characteristic distribution among MASLD and its LF subset versus non-MASLD participants.

Compared to non-MASLD samples, the MASLD group had a lower proportion of females (20.3%vs.29.3%), was older (56vs.51 years), had a higher proportion of BMI > 30 (42.2%vs.28.9%), a lower proportion of never smokers (28.1%vs.36.2%), higher prevalence of diabetes (15.6%vs.7.3%), elevated levels of ALT (20vs.15), AST (20vs.18), total cholesterol (4.76vs.4.68), and hs-CRP (2.84vs.1.33). The detailed comparison between MASLD and non-MASLD participants revealed significant differences in age, gender, BMI, smoking status, diabetes, ALT, AST, total cholesterol, and hs-CRP levels (*P < *.05). Focusing on the subset of MASLD patients with significant fibrosis versus those without, statistically significant differences were again observed in age, BMI, smoking status, diabetes, and hs-CRP levels (*P < *.05).

### Critical Role of High-Sensitivity C-reactive Protein in Metabolic Dysfunction-Associated Steatotic Liver Disease Risk

**Figure 2.**
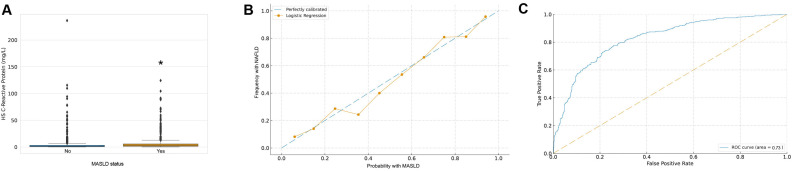
Application of hs-CRP in MASLD Risk Assessment. Note: (A) Distribution of hs-CRP. The box plot reveals the distribution of hs-CRP among participants in both MASLD and non-MASLD groups. The difference between “No” and “Yes” is statistically significant (*P < .05). (B) Calibration curve of the predictive model. The calibration curve displays the relationship between the probability of MASLD occurrence predicted by the model and the actual observed probability. (C) ROC curve analysis. ROC curve reflects the relationship between the model’s true positive rate (sensitivity) and false positive rate (1-specificity) at different decision thresholds.

The study was furthered to examine the biological background of MASLD and its potential association with hs-CRP. Initial observational analysis unveiled a significant discrepancy in the distribution of hs-CRP concentrations between the 2 groups of individuals with and without MASLD, with the MASLD patient group demonstrating higher mean concentrations and distribution variability of hs-CRP (Figure 2A). To further understand this phenomenon and investigate other potential covariates, logistic regression analyses were carried out to delineate the possible influences of various physiological and socio-economic variables on MASLD risk.

During the logistic regression analyses, multiple covariates were considered, including race/ethnicity, HDL-C, gender, age, educational level, smoking status, moderate recreational activities, hs-CRP, total cholesterol, diabetes, ALT and AST levels, and BMI, to ensure the analyses could reveal the relationships between these variables and MASLD risk as accurately as possible. To validate the model’s predictive accuracy and reliability, the model was evaluated through calibration curves (Figure 2B) and ROC curves (Figure 2C). The calibration curve demonstrated fairly accurate predictive capability of the model overall, despite some specific predictive probability intervals exhibiting certain deviations. Meanwhile, the AUC was 0.73, indicating a certain superiority of the model in distinguishing MASLD risk.

Through in-depth statistical analyses and model validation, it can be preliminarily hypothesized that hs-CRP has a certain association with the occurrence of MASLD, and the positive correlation exhibited in the model highlights its significance in predicting MASLD risk.

### Associations Between Discrete High-Sensitivity C-reactive Protein Level Categories and Metabolic Dysfunction-Associated Steatotic Liver Disease Risk

To examine the risk of contracting MASLD, a commonly utilized inflammation marker, hs-CRP, was focused on and its correlation with MASLD and associated fibrosis risk was determined. The data of 4029 participants were meticulously analyzed with a segment manifesting MASLD (n = 1820).

**Figure 3.**
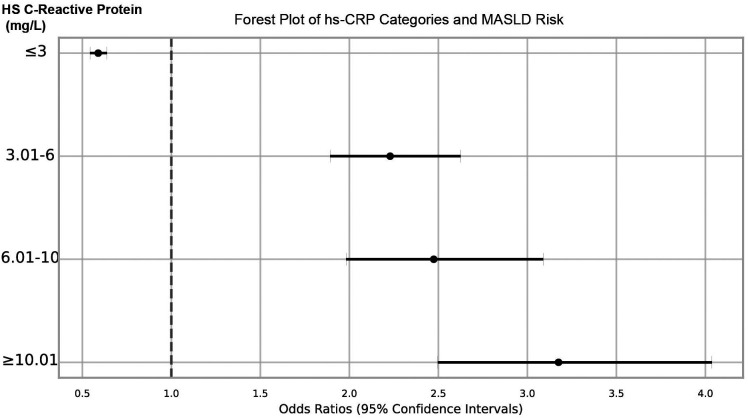
Risk of MASLD at Different hs-CRP Levels. Note: The figure shows the OR and their 95% CI for having MASLD at different levels of hs-CRP. Hs-CRP levels are categorized into 4 groups: ≤ 3 mg/L, 3.01-6 mg/L, 6.01-10 mg/L, and ≥ 10.01 mg/L, with the risk of the group without MASLD serving as the reference group. ORs are calculated through a multivariate logistic regression model and adjusted for other baseline characteristics of the patients. Dots represent ORs, and horizontal lines represent 95% of CIs.

Participants were categorized into 4 distinct categories by subdividing the concentrations of hs-CRP: ≤3 mg/L, (3.01-6) mg/L, (6.01-10) mg/L, and ≥10.01 mg/L. This categorization aimed to further reveal the relative risk of contracting MASLD at different hs-CRP scores. After logistic regression analyses, the OR and their 95% CI for each hs-CRP category were obtained, providing the the relative risk of LF or MASLD compared to the reference category, intending to measure the relationships between hs-CRP concentrations and MASLD risk as precisely as possible. In summary, the OR and 95% CI for MASLD risk at hs-CRP ≤ 3 mg/L were 0.589 (0.543-0.638), at hs-CRP (3.01-6) mg/L were 2.229 (1.892-2.625), at hs-CRP (6.01-10) mg/L were 2.474 (1.982-3.090), and at hs-CRP ≥ 10.01 mg/L were 3.175 (2.497-4.037). Based on the data and the associated forest plot (Figure 3) and the result that the OR for hs-CRP ≤ 3 mg/L was less than 1, it was evident that the risk of MASLD was reduced in this category. The OR was greater than 1 for other hs-CRP categories, indicating an increased risk of MASLD. These data suggested a significantly positive correlation between higher hs-CRP concentrations and increased MASLD risk.

### High-Sensitivity C-Reactive Protein and Liver Fibrosis Risk in Metabolic Dysfunction-Associated Steatotic Liver Disease Patients

**Figure 4.**
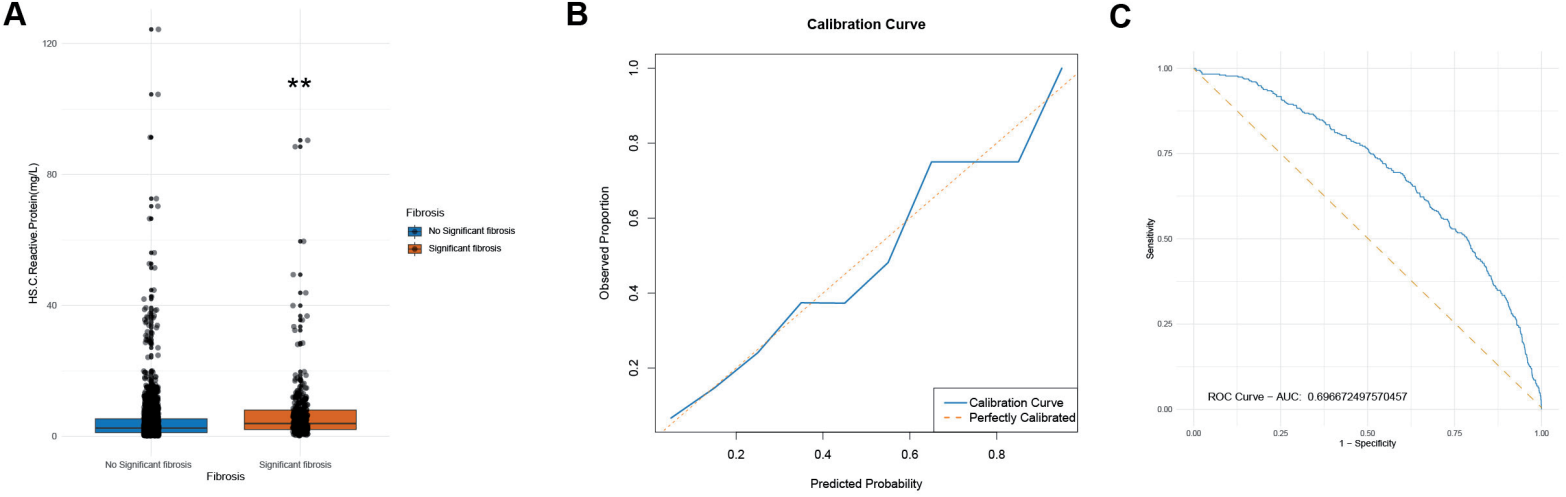
Distribution of hs-CRP in MASLD Patients and Its Ability to Predict LF. Note: (A) Comparison of hs-CRP distribution in MASLD patients. The box plot reflects the distribution of hs-CRP in MASLD patient populations with and without LF. The difference between “No Significant Fibrosis” and “Significant Fibrosis” is statistically significant (**P < .01). (B) Calibration curve of the predictive model. The calibration curve demonstrates the concordance between the probability of LF occurrence predicted by the logistic regression model and the actual observed probability. (C) ROC curve. ROC curve illustrates the sensitivity and 1-specificity of the model at different thresholds.

The potential correlation between hs-CRP and LF was focused on, particularly examining the distribution and strength of this association across different patient groups. The Wilcoxon test indicated a significant difference between hs-CRP levels and LF. Specifically, the Wilcoxon test results demonstrated a significant disparity in hs-CRP distributions between individuals with and without LF (*P* < .01) (Figure 4A). This finding highlights the notable association between hs-CRP and LF and suggests that hs-CRP may play a role in predicting or identifying patients at risk for LF.

Additional insights were derived from further logistic regression analysis. The analysis of ROC curves (Figure 4C) indicated that the model’s AUC value was 0.697. This value indicated a moderate capacity to distinguish individuals with LF from those without, clearly outperforming random prediction. The calibration curve (Figure 4B) closely approximated the 45° line, indicating the reliability of the model’s predictions and that the predicted probabilities accurately reflected the observed incidence rates.

### Association of Varying High-Sensitivity C-Reactive Protein Levels with the Risk of Liver Fibrosis in Patients with Metabolic Dysfunction-Associated Steatotic Liver Disease

**Figure 5.**
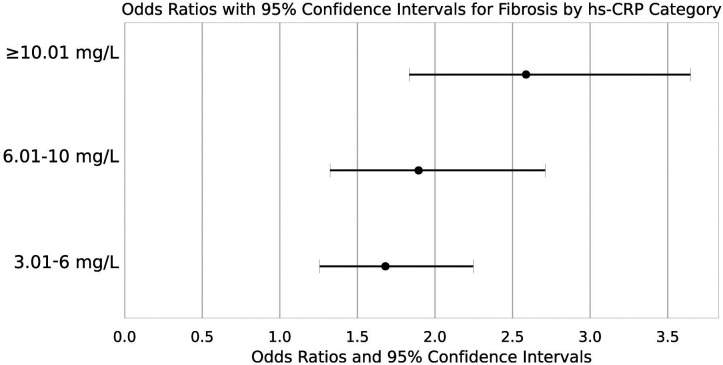
Risk of MASLD Concurrent with Fibrosis at Different hs-CRP Levels. Note: The forest plot compares LF risk across hs-CRP categories (≤ 3 mg/L, 3.01-6 mg/L, 6.01-10 mg/L, ≥ 10.01 mg/L). Each line represents an hs-CRP category, compared with the reference group of hs-CRP ≤ 3 mg/L; dots represent the OR for each category, and horizontal lines represent the 95% CI for each category.

Finally, the association between various ranges of hs-CRP and the risk of LF in patients with MASLD was clarified. The analysis unveiled a prominent relationship between different hs-CRP levels and the risk of LF, as depicted in Figure 5. Compared to individuals with hs-CRP levels ≤3 mg/L, those with hs-CRP levels between 3.01-6 mg/L had a 1.681-fold increased risk of LF (95% CI: 1.257-2.247). The risk further increased to 1.895-fold (95% CI: 1.324-2.712) for individuals with hs-CRP levels of 6.01-10 mg/L. For those with hs-CRP levels ≥10.01 mg/L, the risk of LF significantly rose to 2.587-fold (95% CI: 1.836-3.646). These results suggest that hs-CRP has potential value in stratifying LF risk and monitoring its progression in MASLD patients.

## Discussion

High-sensitivity C-reactive protein is a highly sensitive inflammatory marker produced by the liver.^19^ Unlike the relatively low and stable levels observed in chronic inflammation, acute inflammation triggers a rapid and significant increase in hs-CRP levels. Extensive research has established that inflammatory reactions are crucial in the progression of atherosclerotic cardiovascular disorders, including acute coronary syndrome.^20^ During acute cardiovascular events like myocardial infarction, hs-CRP levels increase sharply in response to coronary artery occlusion, promoting the formation of atherosclerotic plaques and obstructive thrombi.^21^ Additionally, high hs-CRP levels are associated with dismal outcomes in individuals with ischemic stroke.^22^ These characteristics make hs-CRP a widely used and important biomarker for assessing inflammation and associated disease risks.

Non-alcoholic fatty liver disease was historically understood as the accumulation of excess fat in liver cells, which was not caused by alcohol. Typically, the liver contains a small amount of fat, but when more than 5% of liver cells exhibit macrovesicular steatosis, it is classified as fatty liver.^23^ Due to its close association with metabolic diseases, NAFLD has been reclassified as MASLD.^3^ The more severe form of MASLD is known as metabolic dysfunction-associated steatohepatitis, characterized by liver inflammation and damage. As MASLD progresses, it can lead to cirrhosis and hepatocellular carcinoma. The pathogenesis of MASLD involves complex metabolic, inflammatory, and cellular stress processes.^24^ Hepatic fat accumulation activates the immune system, leading to leukocyte aggregation and the production of inflammatory cytokines such as tumor necrosis factor-α and interleukin (IL)-6.^25^ These cytokines stimulate the liver to produce more CRP, thereby elevating hs-CRP levels. High hs-CRP levels are often associated with insulin resistance and metabolic syndrome.^26^ The development of insulin resistance contributes to the enhanced accumulation of fatty acids in the liver, a key factor in the progression of MASLD.^27^ Such metabolic disturbances are likely to provoke ongoing inflammatory responses, leading to a rise in hs-CRP levels. Oxidative stress in the liver of MASLD patients causes oxidative damage to cell membranes and exacerbates inflammation.^28^ This oxidative stress activates inflammatory signaling pathways, resulting in increased hs-CRP synthesis.^29^ While elevated hs-CRP is likely stimulated by these conditions, its role in exacerbating disease progression through inflammatory pathways cannot be excluded. The investigation established a marked positive relationship between hs-CRP levels and the risk for MASLD. Higher levels of hs-CRP were associated with an elevated risk of MASLD, supporting its role as a biomarker of systemic inflammation resulting from metabolic dysfunction. The association was particularly marked between hs-CRP levels within the ranges of 6.01-10 mg/L and those above 10.01 mg/L and the risk for MASLD. This finding highlights the critical predictive value of serum hs-CRP levels in assessing MASLD risk.

Previous research has established that elevated hs-CRP levels are connected with an increased risk of MASLD.^30^ ^31^ These studies, however, left the clinical implications of such elevated hs-CRP levels unspecified. Jamialahmadi et al^32^ found an association between hs-CRP, LF, and steatosis, suggesting that hs-CRP has predictive value for histological liver damage in MASLD patients. They identified a cutoff value of 7 mg/L for hs-CRP, which demonstrated reasonable specificity (76%) for detecting biopsy-confirmed fibrosis and steatosis. Furthermore, the study’s AUC value exceeded that of Jamialahmadi et al,^32^ indicating that this model offers superior accuracy. In contrast to previous research, this study found a pronounced association between hs-CRP levels and MASLD and LF only when hs-CRP levels exceeded 10.01 mg/L. This incremental relationship between hs-CRP levels and LF risk provides a basis for further exploration of its role in MASLD management and intervention. The findings enhance the understanding of the clinical prognostic significance of elevated hs-CRP levels, thus increasing its clinical utility.

Other investigations have highlighted various inflammatory indicators, including Vascular Cell Adhesion Molecule-1 (VCAM1), IL-6, IL-8, and chemokines, which surpass hs-CRP in their ability to differentiate LF.^33^ However, detecting these markers is expensive and not commonly used in clinical practice. This study utilized large sample data to elucidate the positive correlation between hs-CRP levels and patient clinical outcomes. Considering the practicality, ease, and lower cost of hs-CRP detection compared to existing inflammatory markers, hs-CRP holds significant clinical relevance for diagnosing and monitoring NAFLD patients, providing valuable guidance for clinical practice.

Despite the strengths of the study, several limitations should be noted. First, the cross-sectional design limits the ability to establish causality between hs-CRP levels and LF. Future prospective studies are needed to explore these relationships further. Additionally, the exclusion of liver diseases caused by other factors such as HIV infection, iron overload, autoimmune hepatitis, and primary biliary cholangitis might have introduced selection bias, as these were not fully accounted for in the NHANES dataset. Unadjusted potential confounders may also affect the accuracy of the results. Finally, the lack of standardized diagnostic thresholds for MASLD and LF using transient elastography limits the comparability of the findings with other studies that rely on liver histology. Further research is needed to address these limitations and establish specific hs-CRP thresholds for risk stratification.

**Figure 6.**
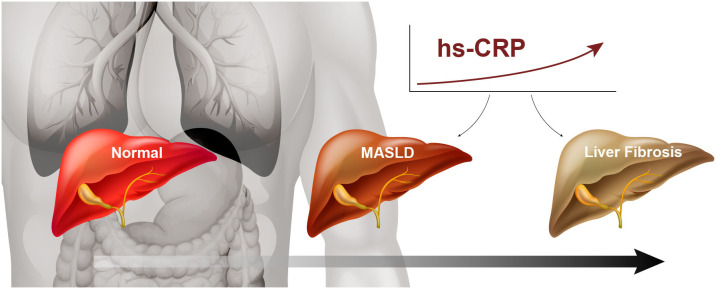
Schematic of the study on the association of hs-CRP levels with MASLD and the risk of LF.

In conclusion, this study demonstrates the association between hs-CRP levels, the occurrence of MASLD, and the development of LF in MASLD patients (Figure 6). Elevated hs-CRP levels are significantly associated with an increased risk of MASLD and LF. These findings highlight the potential of hs-CRP as a biomarker for early diagnosis and risk assessment in clinical settings. Although hs-CRP levels likely reflects systemic inflammation caused by metabolic dysfunction, its potential contribution to disease progression warrants further investigation. Future studies could explore the integration of hs-CRP into preventive detection strategies and the development of targeted therapies to manage inflammation and mitigate disease progression.

## Figures and Tables

**Table 1.** Characteristics for Total Sample and by Disease Status

Characteristics	Overall	MASLD		*P*	Significant Fibrosis		*P*
		Yes	No		Yes	No	
Number	4029.00	1820	2209		350	1470	
Gender, n (%)							.169
Male	2031 (50.4%)	1001 (24.8%)	1030 (25.6%)		204 (11.2%)	797 (43.8%)	
Female	1998 (49.6%)	819 (20.3%)	1179 (29.3%)		146 (8%)	673 (37%)	
Age (years), median (IQR)	54 (38, 66)	56 (43, 66)	51 (35, 66)	.000	58 (47, 67)	56 (42, 66)	.009
Education level, n (%)				.474			.067
<high school	775 (19.2%)	364 (9%)	411 (10.2%)		64 (3.5%)	300 (16.5%)	
high school	908 (22.5%)	400 (9.9%)	508 (12.6%)		93 (5.1%)	307 (16.9%)	
>high school	2346 (58.2%)	1056 (26.2%)	1290 (32%)		193 (10.6%)	863 (47.4%)	
Smoking status, n (%)				.000			.000
Current	498 (12.4%)	188 (4.7%)	310 (7.7%)		26 (1.4%)	162 (8.9%)	
Never	2591 (64.3%)	1133 (28.1%)	1458 (36.2%)		200 (11%)	933 (51.3%)	
Former	940 (23.3%)	499 (12.4%)	441 (10.9%)		124 (6.8%)	375 (20.6%)	
Moderate recreational activities, n (%)				.337			.846
Yes	2331 (57.9%)	1038 (25.8%)	1293 (32.1%)		198 (10.9%)	840 (46.2%)	
No	1698 (42.1%)	782 (19.4%)	916 (22.7%)		152 (8.4%)	630 (34.6%)	
Hs C-Reactive protein (mg/L), median (IQR)	1.93 (0.85, 4.39)	2.84 (1.26, 5.845)	1.33 (0.64, 3.24)	.000	3.9 (2.0675, 8.0175)	2.535 (1.1525, 5.345)	.000
Hs C-Reactive Protein group, n (%)				.000			.000
≤3	2560 (63.5%)	949 (23.6%)	1611 (40%)		136 (7.5%)	813 (44.7%)	
3.01-6	770 (19.1%)	436 (10.8%)	334 (8.3%)		96 (5.3%)	340 (18.7%)	
6.01-10	369 (9.2%)	220 (5.5%)	149 (3.7%)		53 (2.9%)	167 (9.2%)	
≥10.01	330 (8.2%)	215 (5.3%)	115 (2.9%)		65 (3.6%)	150 (8.2%)	
Total cholesterol (mmol/L), median (IQR)	4.73 (4.06, 5.43)	4.76 (4.09, 5.48)	4.68 (4.03, 5.4)	.042	4.55 (3.9, 5.25)	4.825 (4.145, 5.51)	.000
Diabetes, n (%)				.000			.000
Yes	924 (22.9%)	629 (15.6%)	295 (7.3%)		191 (10.5%)	438 (24.1%)	
No	3105 (77.1%)	1191 (29.6%)	1914 (47.5%)		159 (8.7%)	1032 (56.7%)	
ALT (IU/L), median (IQR)	17 (13, 25)	20 (15, 29)	15 (12, 21)	.000	22 (15.25, 34.75)	20 (15, 28)	.001
AST (IU/L), median (IQR)	19 (16, 23)	20 (16, 24)	18 (16, 22)	.000	21 (17, 27.75)	19 (16, 24)	.000
BMI group, n (%)				.000			.000
<25	1019 (25.3%)	141 (3.5%)	878 (21.8%)		6 (0.3%)	135 (7.4%)	
25-30	1310 (32.5%)	514 (12.8%)	796 (19.8%)		38 (2.1%)	476 (26.2%)	
≥30	1700 (42.2%)	1165 (28.9%)	535 (13.3%)		306 (16.8%)	859 (47.2%)	

## Data Availability

The original contributions presented in the study are included in the article/supplementary materials, further inquiries can be directed to the corresponding author.
